# Intensity- and time-matched acute interval and continuous endurance exercise similarly induce an anti-inflammatory environment in recreationally active runners: focus on PD-1 expression in T_regs_ and the IL-6/IL-10 axis

**DOI:** 10.1007/s00421-023-05251-y

**Published:** 2023-06-19

**Authors:** Sebastian Proschinger, Alexander Schenk, Inga Weßels, Lars Donath, Ludwig Rappelt, Alan J. Metcalfe, Philipp Zimmer

**Affiliations:** 1 Division of Performance and Health (Sports Medicine), TU Dortmund University, Institute for Sport and Sport Science, 44227 Dortmund, Germany; 2 Faculty of Medicine, RWTH Aachen University, Institute of Immunology, 52074 Aachen, Germany; 3 Department of Intervention Research in Exercise Training, German Sport University Cologne, Cologne, Germany; 4 Department for Molecular and Cellular Sports Medicine, Institute of Cardiovascular Research and Sports Medicine, German Sport University Cologne, Cologne, Germany

**Keywords:** Regulatory T cells, PD-1, Cytokines, HIIT

## Abstract

**Purpose:**

Acute exercise elicits a transient anti-inflammatory state during the early recovery period. Since recent studies reported on regimen-specific effects on immune-related humoral factors and cellular subsets, this study compared the effects of intensity- and time-matched acute interval and continuous exercise on peripheral anti-inflammatory cellular and humoral immune parameters with a particular focus on the PD-1 expression in CD4^+^ regulatory T cells (T_regs_).

**Methods:**

Twenty-four recreationally active runners (age: 29.7 ± 4.3 years, BMI: 22.2 ± 2.4, VO_2peak_: 56.6 ± 6.4 ml × kg^−1^ × min^−1^) participated in this crossover RCT. Each subject conducted a moderate continuous (MCE) and a high-intensity interval exercise (HIIE) session in a counterbalanced design. Blood was drawn before, immediately after, and 1 h after exercise. T_reg_ subsets and levels of PD-1 and Foxp3 were assessed by flow cytometry. Serum levels of IL-10 and IL-6 were quantified by ELISA.

**Results:**

PD-1 levels on T_regs_ increased within the recovery period after HIIE (*p < *.001) and MCE (*p <  *0.001). Total counts of T_regs_ (HIIE: *p = *0.044; MCE: *p = *.021), naïve T_regs_ (HIIE: *p  <* 0.001; MCE: *p  <* 0.001), and PD-1^+^ effector T_regs_ (eT_regs_) (HIIE: *p = *.002) decreased 1 h after exercise. IL-10 increased 1 h after HIIE (*p < *0.001) and MCE (*p = *0.018), while IL-6 increased immediately after both HIIE (*p = *0.031) and MCE (*p = *0.021). Correlations between changes in IL-6 and IL-10 (*p = *0.017, *r = *0.379) and baseline VO_2peak_ and T_reg_ frequency (*p = *0.002, *r = *0.660) were identified.

**Conclusion:**

This is the first study that investigates PD-1 expression in circulating T_regs_ after acute exercise, revealing an increase in PD-1 levels on eT_regs_ during the early recovery period after intensity- and time-matched HIIE and MCE. Future studies are needed to investigate the PD-1 signalosome in eT_regs_, together with the expression of key effector molecules (i.e., IL-10, TGF-β, IL-35, CTLA-4) to elucidate PD-1-dependent changes in cellular function. Based on changes in serum cytokines, this study further reveals a regimen-independent establishment of an anti-inflammatory milieu and underpins the role of the IL-6/IL-10 axis.

**Supplementary Information:**

The online version contains supplementary material available at 10.1007/s00421-023-05251-y.

## Introduction

Acute physical exercise represents a physiological stressor that elicits a proinflammatory response, characterized by increases in humoral (i.e., proinflammatory cytokines) and cellular immune parameters (i.e., immune cells with effector functions) (Hoffman-Goetz and Pedersen 1994). This is followed by a shift toward an enhanced anti-inflammatory environment in the periphery through a compensatory secretion of anti-inflammatory cytokines and the predominance of immune cells with anti-inflammatory characteristics (Petersen and Pedersen 2005; Gebhardt and Krüger 2022). These changes are largely affected by exercise intensity and duration, with higher intensities and prolonged bouts provoking stronger alterations in pro- and anti-inflammatory mediators (Cabral-Santos et al. 2019; Cerqueira et al. 2020). High-intensity interval exercise (HIIE), characterized by short intense bouts interspersed by periods of active or passive recovery, represents a widely accepted time-efficient alternative to moderate continuous exercise (MCE) (Bartlett et al. 2011; Milanović et al. 2015). A growing number of studies have focused on the differential effect of acute HIIE and MCE on both cytokines and immune cell subsets with some studies reporting regimen-specific effects (Leggate et al. 2010; Krüger et al. 2016; Antunes et al. 2019; Wadley et al. 2020). Alterations in the cellular anti-inflammatory compartment, which usually focus on CD4^+^ regulatory T cell (T_reg_), are less consistent across studies (Proschinger et al. 2021) compared to changes in humoral factors such as interleukin (IL)-10 and IL-6 (Fischer 2006; Cabral-Santos et al. 2019; Cerqueira et al. 2020). It is assumed that T_regs_ play a role in the temporary anti-inflammatory state after exercise and might contribute to the rise in serum IL-10 levels which represents a hallmark in the post-exercise anti-inflammatory response (Cabral-Santos et al. 2019). Seminal work from Steensberg et al. revealed a major contribution of IL-6 to increase IL-10 serum levels by infusing recombinant human IL-6 corresponding to the levels obtained during strenuous exercise (Steensberg et al. 2003), thereby proposing the concept of IL-6-mediated release of IL-10 into the bloodstream.

The programmed cell death protein 1 (PD-1) is highly expressed on T_regs_ with an effector phenotype showing enhanced immunosuppressive functions and has further been shown to promote T_reg_ differentiation, phenotypic stability, functional capacity, and expansion (Francisco et al. 2009; Cai et al. 2019; Ohue and Nishikawa 2019). However, compelling evidence from recent human and animal studies in cancer, autoimmunity, and infection increasingly demonstrate a role of PD-1 signaling to hamper T_reg_ suppressive capacity (Kamada et al. 2019; Kumagai et al. 2020; Tan et al. 2021; Perry et al. 2022). This strongly suggests an inhibition of T_reg_ function after binding of PD-1 to its ligands (i.e., programmed death-ligand 1 (PD-L1)).

Taken together, the aim of this randomized controlled crossover study is to compare the effects of intensity- and time-matched acute HIIE and MCE on humoral (i.e., IL-10 and IL-6) and cellular (i.e., T_regs_) immune parameters that are generally associated with an anti-inflammatory environment in the early recovery period after exercise. The effect of exercise on PD-1 expressing T_regs_ has not been conducted so far and should provide a more detailed insight into their potential contribution to the exercise-induced anti-inflammatory milieu within the bloodstream.

## Methods

### Subjects

A total of 12 male and 12 female healthy (defined as body mass index < 30), recreationally active runners (classified as running between 2 and 5 h per week) between 18 and 35 years of age participated in this randomized controlled crossover study. Subjects were excluded if they had any previous history of muscle disorder, cardiac or kidney disease, or were taking medication (e.g., anti-inflammatory drugs, antibiotics) or nutritional supplements.

The study was approved by the local ethics committee of the German Sport University Cologne. All subjects signed a written informed consent prior to participation.

### Baseline exercise testing and randomization

Subjects were asked to refrain from caffeine, alcohol, and strenuous exercise for at least 24 h before testing, to arrive fasted (no food intake for at least 2 h) and well hydrated. The baseline testing included a graded exercise test on a treadmill until exhaustion to measure aerobic capacity quantified as peak oxygen uptake (VO_2peak_). In detail, the subjects warmed up for 5 min at 6–8 km/h. Following this, they completed a maximal incremental exercise test to exhaustion which started at a speed of 8 km/h, increasing the speed by 1 km/h every minute until the subjects were not able to maintain the required speed. Thereafter, the subjects were randomized either into group 1 (HIIE-MCE, *n = *10) or group 2 (MCE-HIIE, *n = *14). Concealed randomization was ensured using Randomization-In-Treatment-Arms software (Evident, Germany) with BMI, VO_2peak_, and age as stratification factors.

### High-intensity interval and moderate continuous exercise regimens

**Fig. 1:**
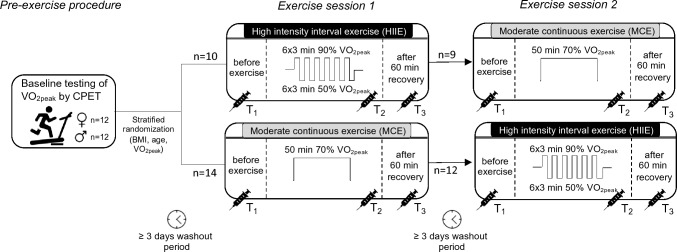
Schematic overview of the study design. *BMI* body mass index, *CPET* cardiopulmonary exercise testing, *VO*_*2peak*_ peak oxygen uptake

Similar to baseline testing, subjects were asked to refrain from caffeine, alcohol, and strenuous exercise for at least 24 h before each intervention, to arrive fasted and well hydrated. At least 3 days after baseline testing, the subjects conducted the first exercise session. Due to the design of the study, neither the subject nor the investigator was blinded to group allocation. After a washout period of at least 3 days, subjects visited the laboratory again to conduct the other exercise session. A washout period of 3 days is considered sufficient, since immunological alterations generally return to baseline levels within 24 h following an acute bout of exercise (Simpson et al. 2015). Both exercise trials were carried out at the same time of the day and were matched for intensity and duration (Bartlett et al. 2012). A detailed depiction of the study design is shown in Fig. 1.

The HIIE session started with a 7-min warm-up at a running speed corresponding to 70% of VO_2peak_, followed by six 3-min intervals of running at a speed corresponding to 90% VO_2peak_. These intervals were interspersed by 3-min active recovery periods at a running speed corresponding to 50% VO_2peak_. The protocol finished with a 7-min cool down at 70% VO_2peak_. The MCE session started at a running speed corresponding to 70% VO_2peak_ which remains constant throughout the 50-min intervention period.

In both exercise sessions, 24 ml of blood was taken from the medial cubital vein and collected in potassium ethylenediaminetetraacetic acid (EDTA)-containing tubes (Vacutainer, BD) before (T_1_), immediately after (T_2_), and 60 min (T_3_) after exercise. Another 8 ml of blood was collected into a serum separation tube (BD SST™ II Advance, BD). During the 1-h recovery period after exercise, any form of physical activity was prohibited.

### Blood preparation

Whole blood, collected in EDTA tubes, was used for peripheral blood mononuclear cell (PBMC) isolation by density gradient centrifugation using a separation medium (Ficoll-Paque^™^ PLUS, Thermo Fisher Scientific). Blood samples were diluted with PBS, carefully layered on top of the separation medium, and centrifuged for 30 min at 800xg in room temperature (RT). After extraction of the PBMC containing interphase, cells were washed with PBS and centrifuged for 10 min at 800xg in RT. The remaining cell pellet was resuspended in Recovery™ cell culture freezing medium (Thermo Fischer Scientific, Waltham, MA, USA), aliquoted, and frozen at − 80 °C overnight. PBMCs were stored at − 150 °C until flow cytometry analysis. Serum tubes were set at RT for 30 min. After clotting, tubes were centrifuged for 10 min at 1600xg in RT. Serum samples were aliquoted and stored at − 80 °C until analysis. Blood cell count analysis was performed from EDTA blood using a hematology analyzer (SYSMEX XN-1000, Norderstedt, Germany). The lymphocyte count was then used to calculate the absolute cell number of peripherally circulating T cells and their subsets according to the cell proportions derived by flow cytometry.

### Flow cytometry

**Fig. 2:**
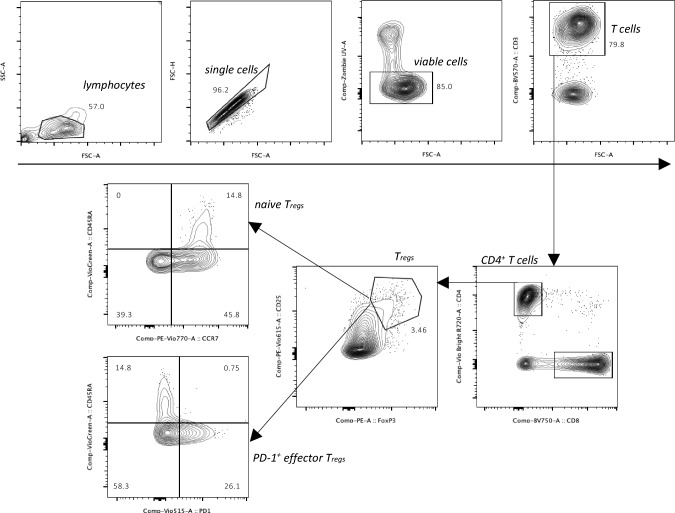
Gating strategy

Flow cytometry analysis was performed using a Cytek^®^ Aurora full spectrum flow cytometer (Cytek Biosciences, California, USA). Cryopreserved PBMCs were gently thawed with a mean recovery of 87.50% viable cells, assessed by the Zombie UV™ Fixable Viability Kit (BioLegend, BioLegend, San Diego, CA, USA). 0.5 × 10^6^ PBMCs were stained in duplicate by using anti-CD3 *BV570* (UCHT1), anti-CD4 *VioBright R720* (REA623), anti-CD25 *PE-Vio615* (REA570), anti-CD45RA *VioGreen* (REA1047), anti-CD197 (CCR7) *PE-Vio770* (REA108), anti-CD279 (PD-1) *VioBright 515* (REA1165), and anti-FoxP3 *PE* (REA1253) (Miltenyi Biotec, Bergisch Gladbach, Germany). The Transcription Factor Staining Buffer Set (Miltenyi Biotec, Bergisch Gladbach, Germany) was used for nuclear staining. Isotype controls were used to rule out unspecific binding of antibodies. Gating was performed using FlowJo^™^ 10.8.1 (see Fig. 2 for gating strategy). T_regs_ were gated as CD4^+^CD25^high^FoxP3^+^, PD-1^+^ effector T_regs_ (eT_regs_) as CD4^+^CD25^high^FoxP3^+^CD45RA^−^PD-1^+^, and naïve T_regs_ (nT_regs_) as CD4^+^CD25^high^FoxP3^+^CD45RA^+^CCR7^+^. Median fluorescence intensity (MFI) was calculated in FlowJo™ and used as a relative surrogate marker of protein level.

### ELISA

Serum concentrations of interleukin IL-10 and IL-6 were quantified using OptEIA assays from BD Pharmingen (Heidelberg, Germany) according to the manufacturer´s instructions.

### Statistics

Normal distribution was assessed via the Shapiro–Wilk test. Outliers were detected by winsorization at 1% and 99.87% (mean ± 3 standard deviation) and excluded from analysis. The mixed model for repeated measures (MMRM) was used for data analysis. Using this technique, no imputation was required, and all data was taken into account. Repeated measures mixed model tests were controlled for baseline covariance (Overall and Doyle 1994). In case of statistical main effects for time and/or interaction (time × group), Bonferroni-corrected pairwise comparisons were applied to determine within- and/or between-group differences. Pearson's bivariate correlation was used to evaluate the relationship between delta changes of IL-10 and IL-6 levels. Spearman's rank order correlation was used to evaluate the relationship between baseline VO_2peak_ and T_reg_ frequency. The level of significance was set at *p* ≤ 0.05. All data are presented as mean ± standard error of the mean (SEM). SPSS version 28 (IBM^®^, Armonk, NY, USA) was used for statistical analysis. GraphPad PRISM v.9 was used for graphical illustration.

## Results

All 24 randomized subjects completed both exercise sessions. Samples from one session were missing for three subjects, leading to the total analysis of *n = *45. Baseline characteristics of anthropometric and performance data, separated by training sequence (HIIE-MCE vs. MCE-HIIE), are shown in Table 1. Detailed MMRM results for all outcome measurements, including raw data, are provided in Table S1.

**Table 1:** Baseline characteristics of study participants separated by exercise sequence

Parameter	HIIE-MCE (*n = *10)	MCE-HIIE (*n = *14)
Age [y]	30.0 ± 3.9	29.4 ± 4.7
Height [cm]	175.6 ± 6.4	177.9 ± 9.5
Weight [kg]	68.8 ± 7.5	70.3 ± 13.5
BMI [kg/m^2^]	22.2 ± 1.30	22.2 ± 3
Gender (male/female)	5/5	7/7
Smoking status (yes/no)	0/10	0/14
VO_2peak_ [ml × kg^−1^ × min^−1^]	56.8 ± 7.1	56.6 ± 6.2

Data is presented as mean ± SD. *BMI* body mass index, *HIIE* high-intensity interval exercise, *MCE* moderate continuous exercise, *VO*_*2peak*_ peak oxygen consumption

### Changes in T_reg_ cell numbers and frequencies after exercise

**Fig. 3:**
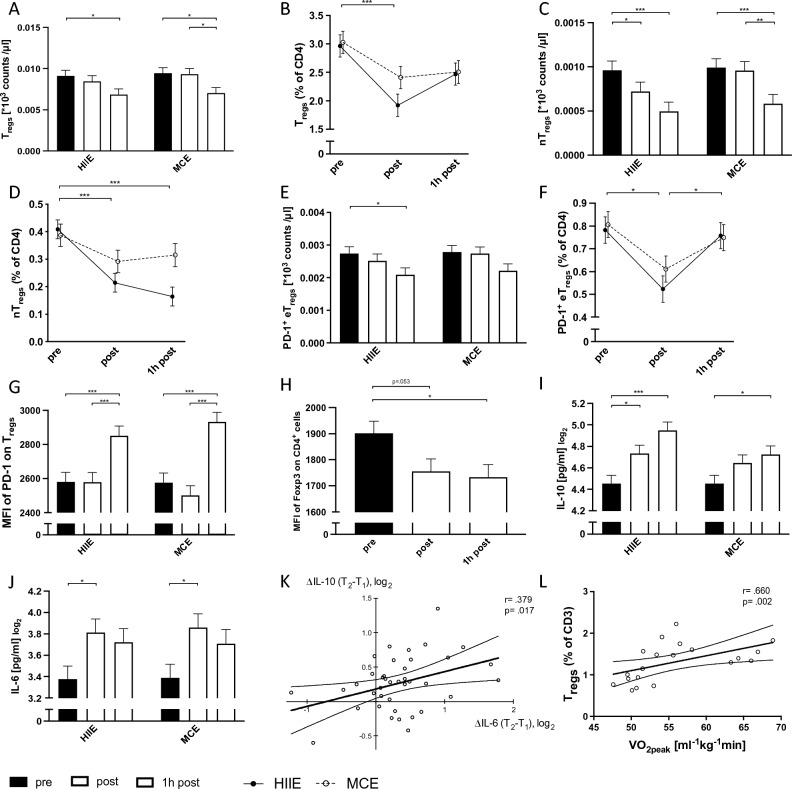
Changes in cell numbers and frequencies of T_regs_ (**A + B**), naïve T_regs_ (**C + D**), PD-1^+^ effector T_regs_ (**E + F**), levels of PD-1 (within T_regs_) (**G**) as well as IL-10 and IL-6 serum levels (**I + J**) are presented for high-intensity interval exercise (HIIE) and moderate continuous exercise (MCE), respectively. Changes in levels of Foxp3 (within CD4^+^ cells) (**H**) are presented as pooled HIIE and MCE data, since only main effects were detected. Correlation analysis are presented between delta changes of IL-6 and IL-10 (**K**) and between VO_2peak_ and T_reg_ frequency (**L**). Data is presented as mean ± SEM. Statistically significant time effects are marked as follows: *0.005 < *p* ≤ 0.05, **0.001 < *p* ≤ 0.005, *** *p* ≤ 0.001. *HIIE* high-intensity interval exercise, *MCE* moderate continuous exercise, *MFI* median fluorescense intesity, *PD-1* programmed cell death protein 1, *T*_*regs*_ regulatory T cells, Δ delta change

A significant time effect was observed for both T_regs_ (*p < *0.001) and nT_regs_ (*p < *0.001) counts which decreased below baseline levels 1 h after HIIE (T_regs_: *p = *0.044; nT_regs_: *p < *0.001) and MCE (T_regs_: *p = *0.021; nT_regs_: *p < *0.001), respectively (Fig. 3, A + C). Further, nT_regs_ decreased immediately after HIIE (*p = *0.009). The frequency of T_regs_ (*p < *0.001) and nT_regs_ (*p < *0.001) within CD4^+^ cells decreased immediately after HIIE, but only nT_regs_ remained below baseline at T_3_ (*p < *0.001) (Fig. 3, B + D). A significant main time effect for PD-1^+^ eT_reg_ cell counts (*p = *0.002) was identified, with post hoc tests revealing a decline 1 h after HIIE (*p = *0.016) (Fig. 3, E). The frequency of PD-1^+^ eT_regs_ declined after HIIE (*p = *0.007) and returned to baseline levels at T_3_ (Fig. 3, F).

### PD-1 MFI within T_regs_ increases during the recovery period above baseline levels, whereas Foxp3 MFI within CD4^+^ T cells decreases after exercise

An increase in PD-1 MFI within Tregs during the recovery period (T_2_ to T_3_) was observed after both regimens (HIIE: *p* < 0.001; MCE: *p* < 0.001), with the levels being higher compared to baseline (HIIE: *p* < 0.001; MCE: *p* < 0.001) (Fig. 3, G). A significant main time effect for Foxp3 MFI within CD4^+^ was identified, with post hoc tests revealing no group-specific time effects (supplement Fig. S1, A), but a decline 1 h after pooled MCE and HIIE data (*p = *0.020) (Fig. 3, H).

### Exercise-induced increase of IL-10 correlates with the increase in IL-6

IL-10 serum levels increased immediately after HIIE (*p = *0.012) and tend to increase further during the recovery period (T_2_ to T_3_: *p = *0.075; T_1_ to T_3_: *p < *0.001) (Fig. 3, I). An increase of IL-10 after MCE was shown 1 h after exercise (*p = *0.018). A significant time effect of IL-6 was revealed, showing an increase immediately after HIIE (*p = *0.031) and (MCE: *p = *0.021), respectively (Fig. 3, J). Positive correlations were identified between delta values of IL-10 and IL-6 with associations between IL-10 ΔT_2_− T_1_ and IL-6 ΔT_2_− T_1_ (*p = *0.017, *r = *0.379) (Fig. 3, K) as well as IL-10 ΔT_3_− T_1_ and IL-6 ΔT_2_− T_1_ (*p = *0.010, *r = *0.418) (Fig. S1, B).

### Baseline VO_2peak_ is positively correlated with T_reg_ frequency

Spearman's rank order correlation revealed a significant positive association between baseline T_regs_ frequency (% of CD3^+^ cells) and VO_2peak_ (*p = *0.002, *r = *0.660) (Fig. 3, L).

## Discussion

By applying intensity- and time-matched acute HIIE and MCE in a randomized controlled crossover design, we provide evidence for an exercise regimen-independent effect on humoral and cellular immune parameters that are generally associated with a temporary anti-inflammatory state after exercise cessation. The decline in T_reg_ cell numbers is accompanied by an increase in PD-1 levels on eT_regs_ as well as a decrease in Foxp3 levels within CD4^+^ T cells. Whether the cellular kinetics in conjunction with the molecular changes are leading to a decrease in T_regs_ functionality needs to be further investigated. Methodological strategies on how to address this question experimentally will be discussed briefly. Further, an enhanced exercise-induced anti-inflammatory environment is characterized by the increase in IL-10 serum levels which is positively correlated with the increase in IL-6. This underpins the "secretory relationship" between both cytokines, originally being described in an earlier study through intravenous administration of recombinant IL-6 (Steensberg et al. 2003), in a real exercise context.

Studies investigating changes in T_reg_ cell counts and frequency after acute exercise revealed heterogeneous results (Proschinger et al. 2021). Here, we observed a decrease of T_reg_ and nT_reg_ counts 1 h after both regimens, whereas the frequency of all T_reg_ subsets within the CD4^+^ T cells decreased immediately after HIIE. This indicates a reduced contribution of T_reg_ subpopulations to the overall CD4^+^ T cell compartment after interval exercise. In regards to changes in cell counts during the recovery period, Krüger et al. observed an increase in T_regs_ in untrained males immediately after 30 min of HIIE which remained elevated 3 h after exercise cessation (Krüger et al. 2016). Although the differences in the subject´s fitness level could serve as a determining factor of the different T_reg_ response, Dorneles et al. revealed no differences in the frequency of T_regs_ in response to acute HIIE between subjects with high and low physical fitness (Dorneles et al. 2019). With regard to predicting the cellular function based on the total amount of circulating cells, it is debatable whether a mere change in a population´s cell count is indicative of a change in the overall functionality. A recent meta-analysis investigating the effect of acute exercise on NK cells revealed that the change in cellular function is not associated with the exercise-induced change in cell counts (Rumpf et al. 2021). This underlines the need to assess the function of T_regs_ either directly or by markers indicative of cellular functionality. Cellular function can be significantly modulated by surface receptor interactions and downstream signaling. The effect of PD-1 activation on T_reg_ functionality has been investigated thoroughly during the recent years with human and animal studies providing strong evidence that PD-1 signaling impairs T_reg_ anti-inflammatory capacity (Kamada et al. 2019; Kumagai et al. 2020; Tan et al. 2021; Perry et al. 2022). Therefore, the rise in PD-1 levels on T_regs_ during the recovery period after both HIIE and MCE observed in this study may be indicative of a compromised T_reg_ function. Since binding of PD-1 to its main ligand PD-L1 is primarily cell contact dependent, it is unclear whether circulating PD-1^+^ eT_regs_ receive a proper inhibitory signal compared to tissue-resident PD-1^+^ eT_regs_ residing in proximity to surface PD-L1-expressing cells. However, it has been shown that PD-L1 can also be secreted as truncated soluble PD-L1 (sPD-L1) which binds to PD-1, inducing downstream inhibitory signaling (Mahoney et al. 2019). Interestingly, Wadley et al. observed increased serum levels of sPD-L1 up to 1 h after both HIIE and MCE with no regimen-specific differences (Wadley et al. 2020). Since their exercise protocols only marginally deviate from those we used, a similar physiological response can be assumed. Future studies are needed to experimentally address whether the exercise-induced rise in PD-1 levels on T_regs_ in conjunction with increased circulating sPD-L1 during the early recovery period downregulates their cellular function through enhanced PD-1 signaling. To better resolve PD-1 pathway activation, T_regs_ need to be purified and analyzed via techniques that are both sensitive and comprehensive enough to infer downstream signaling following receptor activation. This can either be done on the RNA or protein level by methods such as targeted sequencing or mass spectrometry which aims at detecting key factors implicated in PD-1 signaling, i.e., SHP-1, SHP-2, ZAP70, ERK, PI3K, AKT, and PKCθ (Arasanz et al. 2017; Wang et al. 2021). Together with the detection of T_reg_-related effector molecules such as IL-10, TGF-β, IL-35 or CTLA-4, changes in the PD-1 signalosome can be associated with cellular function.

In addition to our findings in T_reg_ cells, Schenk et al. (Schenk et al. 2020) demonstrated a decrease in PD-1 MFI on CD8^+^ T cells 1 h after an acute bout of continuous cycling at 60% peak power output for 50 min, revealing a phenotypically opposing effect in T cells from a different lineage with fundamentally different functions. This imply a cell type-specific effect of endurance exercise on PD-1 surface expression which warrants further investigation.

Our observation that levels of Foxp3—the main defining transcription factor of the T_reg_ cell lineage—within CD4^+^ T cells drops below baseline levels after exercise is indicative of a decline in T_reg_ function by a diminished phenotypic stability and reduced Foxp3 target gene transcription (Colamatteo et al. 2020). By using purified T_regs_, Minuzzi et al. have shown that mRNA expression of Foxp3, IL-10, and TGF-β does not change up to 1 h after exhaustive exercise (Minuzzi et al. 2017) assuming that the T_reg_ phenotype and key transcriptional activities do not change. Albeit gene expression on the transcriptional level may be unchanged, the translation of mRNA transcripts coding for Foxp3 could be downregulated, resulting in lower amounts of Foxp3 proteins (De Sousa Abreu et al. 2009). Another explanation could be specific post-translational modifications of Foxp3 which can result in protein degradation, thereby downregulating its cellular concentration (Colamatteo et al. 2020).

The increase of IL-10 and IL-6 in response to acute exercise is in line with the literature (Fischer 2006; Cabral-Santos et al. 2019), but it has been shown in both cycling (work- and time-matched) and running (time-matched) conditions, that HIIE causes a significantly greater increase in serum IL-6 compared to MCE (Leggate et al. 2010; Sim et al. 2013). The positive association between changes in IL-10 and IL-6 serum levels as shown in this study provides evidence for the IL-6/IL-10 axis in a real exercise context, which originally has been postulated by Steensberg et at. after intravenous administration of recombinant IL-6 levels corresponding to concentration observed during acute exercise (Steensberg et al. 2003). Whereas IL-6 is mainly produced by the working muscle (Febbraio and Pedersen 2002), it is unknown which cell types contribute to the increase in IL-10. Besides T_regs_, type 1 regulatory T cells are another cell type of the CD4^+^ lineage that is known to produce high amounts of IL-10 (Roncarolo et al. 2006), but its role in the acute exercise setting has not been well described yet. It is worth mentioning that IL-10 can be secreted not only by immune cells from the lymphoid lineage, but also from the myeloid lineage and nonhematopoietic cells, thereby posing several candidates that may be involved in the exercise-induced rise in serum IL-10 (Saraiva et al. 2020).

The observation that the cardiorespiratory fitness is positively associated with T_reg_ levels is in line with other studies reporting this association in different cohorts (i.e., elite athletes, healthy older women, pooled fit and unfit men) (Weinhold et al. 2016; Dorneles et al. 2019; Koliamitra et al. 2019), thereby strengthening the available evidence that regular exercise contributes to a more balanced peripheral immune system (Nieman and Wentz 2019). This consistent finding implies that cellular mechanisms between the single exercise bouts are involved, either by promoting the generation and subsequent secretion of thymic T_regs_ or the differentiation of naïve CD4^+^ T cells into peripherally induced T_regs_. A proposed mechanism in this context is an exercise-induced increase in kynurenine, which is taken up by circulating naïve CD4^+^ T cells, binds to the cytosolic aryl hydrocarbon receptor, and activates gene expression required for inducing a T_regs_ phenotype (Joisten et al. 2020). Since HIIE has been shown to be more effective in improving cardiorespiratory fitness (Milanović et al. 2015), future studies may compare the effect between a long term HIIE and MCE training regimen on the frequency of circulating T_regs_ within the T cell compartment.

Taken together, our results reveal an upregulation of surface PD-1 levels on circulating eT_regs_ during the early recovery period after intensity- and time-matched HIIE and MCE. In conjunction with recently published studies (Kamada et al. 2019; Kumagai et al. 2020; Wadley et al. 2020; Tan et al. 2021; Perry et al. 2022), this indicates an activation of inhibitory signaling in T_regs_ that may be potentiated by increased levels of exercise-induced sPD-L1 levels. Therefore, future studies are needed to investigate the PD-1 signaling pathway in depth by using technologies sensitive enough to depict the PD-1 mediated signalosome in conjunction with changes in effector molecule levels in eT_regs_ (i.e., by applying targeted sequencing or mass spectrometry). In addition, this study provides evidence for the IL-6/IL-10 axis in the exercise context which, based on the kinetics of circulating cytokines, leads to an anti-inflammatory state during the early recovery period irrespective of the exercise regimen. It would further be important to consider later time points such as two and four hours post-exercise for both cellular and humoral factors to increase the resolution of the body´s physiological response during the late recovery phase after exercise. Future studies may also consider the contribution of other circulating immune cell types with immunoregulatory functions (i.e., type 1 regulatory T cells or myeloid derived suppressor cells) to the exercise-induced anti-inflammatory response during the early and late recovery period with a main focus on the production of IL-10.

### Strengths and limitations

The strengths of this study comprise the accurate matching of exercise modalities for intensity and duration, respectively. Since this study represents a secondary analysis, no power calculation for the investigated endpoints was performed. Nevertheless, a comparatively large sample size was analyzed in a randomized controlled crossover design. Further studies may benefit from an additional passive control group. However, since all measurement time points were collected within 2 h, a passive control group was not considered as a requirement in this study to control for natural fluctuations in the markers we addressed. Further, a broader experimental setup considering other circulating regulatory immune cells as well as outcomes indicative of direct cellular function (i.e., modulation of signaling pathways, production of effector molecules), especially in eT_regs_, needs to be addressed in further studies. A special focus was placed on the early recovery period within the first hours after acute exercise, since this is the most frequently reported time frame with regard to changes in cellular and humoral immune kinetics. However, further time points that cover the late recovery period provide additional information on changes in cell signaling as well as circulating T_reg_ counts and cytokines harboring anti-inflammatory properties, so that future studies may consider including later time points up to 4–6 h.

## Conclusion

Similar effects of intensity- and time-matched interval and continuous exercise on humoral and cellular parameters that are generally associated with a temporary anti-inflammatory environment were revealed. For the first time, PD-1 expression in T_regs_ was assessed in response to acute exercise showing a rise in surface levels during the early recovery period irrespective of the exercise regimen. Further studies are needed to identify alterations in the PD-1 signalosome in eT_regs_ and whether changes in the downstream signaling are associated with an altered cellular function based on the expression of effector cytokines. Further, the observed correlation between the increase in IL-10 and IL-6 serum levels provides evidence of a previous finding reporting on the IL-6/IL-10 axis in a non-exercise context. In this regard, future studies may focus also on other regulatory immune cells such as type 1 regulatory T cells or myeloid derived suppressor cells to determine their potential contribution to the peripheral anti-inflammatory response when it comes to IL-10 secretion after acute exercise.

### Supplementary Information

Below is the link to the electronic supplementary material.

Supplementary file1 (DOCX 614 KB)

## Data Availability

The original data can be requested from the authors.

## Abbreviations

BMI: Body mass index
EDTA: Ethylenediaminetetraacetic acid
ELISA: Enzyme-linked immunosorbent assay
eT_regs_: Effector T_regs_
HIIE: High-intensity interval exercise
IL: Interleukin
MCE: Moderate continuous exercise
MFI: Median fluorescence intensity
MMRM: Mixed model for repeated measures
nT_regs_: Naïve T_regs_
PBMCs: Peripheral blood mononuclear cells
PD-1: Programmed cell death protein 1
PD-L1: Programmed death-ligand 1
RT: Room temperature
SEM: Standard error of the mean
sPD-L1: Soluble PD-L1
T_regs_: CD4^+^ regulatory T cells
VO_2peak_: Peak oxygen consumption
