# Supplementary material for: Intensity- and time-matched acute interval and continuous endurance exercise similarly induce an anti-inflammatory environment in recreationally active runners: focus on PD-1 expression in Tregs and the IL-6/IL-10 axis
Source: Eur J Appl Physiol. 2023 Jun 19;123(11):2575–84. doi: 10.1007/s00421-023-05251-y (PMC10615943; doi:10.1007/s00421-023-05251-y)
Supplement: Supplementary file 1 — Supplementary file1 (DOCX 614 KB) [file 421_2023_5251_MOESM1_ESM.docx]

Title

Intensity- and time-matched acute interval and continuous endurance exercise similarly induce an anti-inflammatory environment in recreationally active runners: focus on T_regs_ and the IL-6/IL-10 axis

Journal

European Journal of Applied Physiology

Authors and affiliations

Sebastian Proschinger^1^, Alexander Schenk^1^, Inga Weßels^2^, Lars Donath^3^, Ludwig Rappelt^3^, Alan J. Metcalfe^4^, Philipp Zimmer^1^

^1^TU Dortmund University, Institute for Sport and Sport Science, Division of Performance and Health (Sports Medicine), 44227 Dortmund, Germany

^2^RWTH Aachen University, Institute of Immunology, Faculty of Medicine, 52074 Aachen, Germany

^3^Department of Intervention Research in Exercise Training, German Sport University Cologne, Cologne, Germany

^4^Department for Molecular and Cellular Sports Medicine, Institute of Cardiovascular Research and Sports Medicine, German Sport University Cologne, Cologne, Germany

Corresponding author: Prof. Philipp Zimmer, philipp.zimmer@tu-dortmund.de

**Fig. S1** Changes in levels of Foxp3 (within CD4^+^ cells) (**A**) are presented for high-intensity interval exercise (HIIE) and moderate continuous exercise (MCE), respectively. Correlation analysis are presented between delta changes of IL-6 (ΔT2-T1) and IL-10 (ΔT3-T1) (**B**). Data is presented as mean ± SEM. HIIE: high-intensity interval exercise, MCE: moderate continuous exercise, MFI: median fluorescense intesity, T_1_: pre exercise, T_2_: immediately post exercise, T_3_: one hour post exercise

**Tab. S1** Detailed Mixed Model or Repeated Measures results separated by group and main effects for reported outcome measures.

|  |  | Group | |  | Main effects | | | |
| --- | --- | --- | --- | --- | --- | --- | --- | --- |
| Outcome |  | *MCE* | *HIIE* |  |  | *Time* | *Group* | *Time x Group* |
| MFI PD-1 on T_regs_ |  |  |  |  |  |  |  |  |
|  | Pre | 2576.9 ± 55.3 | 2579.7 ± 56.5 |  |  |  |  |  |
|  | Post | 2502.6 ± 55.3 | 2579.2 ± 56.5 |  | F-value: | 31.109 | 0.00003 | 1.292 |
|  | 1h post | 2932.3 ± 56.4*** | 2851.9 ± 56.5*** |  | p-value: | **<0.001** | 0.996 | 0.280 |
| MFI Foxp3 on CD4^+^ |  |  |  |  |  |  |  |  |
|  | Pre | 1903.106 ± 66.1 | 1898.218 ± 67.5 |  |  |  |  |  |
|  | Post | 1768.28 ± 66 | 1742.968 ± 67.5 |  | F-value: | 4.573 | 0.0002 | 0.098 |
|  | 1h post | 1719.579 ± 67.4 | 1747.127 ± 67.5 |  | p-value: | **0.013** | 0.989 | 0.907 |
| MFI Foxp3 on CD4^+^ |  |  |  |  |  |  |  |  |
| *pooled* | Pre | 1900662 ± 47.2 | |  |  |  |  |  |
|  | Post | 1755.624 ± 47.2 | |  |  |  |  |  |
|  | 1h post | 1733.353 ± 47.7* | |  |  |  |  |  |
| T_regs_ [*10^6^/μl] |  |  |  |  |  |  |  |  |
|  | Pre | 9.464 ± 0.654 | 9.114 ± 0.682 |  |  |  |  |  |
|  | Post | 9.342 ± 0.666 | 8.446± 0.682 |  | F-value: | 7.882 | 0.606 | 0.174 |
|  | 1h post | 7.039 ± 0.666* | 6.856± 0.682* |  | p-value: | **<0.001** | 0.441 | 0.841 |
| T_regs_ [% CD4^+^] |  |  |  |  |  |  |  |  |
|  | Pre | 3.027 ± 0.196 | 2.963 ± 0.196 |  |  |  |  |  |
|  | Post | 2.410 ± 0.196 | 1.919 ± 0.196* |  | F-value: | 9.017 | 1.573 | 0.829 |
|  | 1h post | 2.507 ± 0.200 | 2.468 ± 0.196 |  | p-value: | **<0.001** | 0.217 | 0.440 |
| PD1^+^ eT_regs_ [*10^6^/μl] |  |  |  |  |  |  |  |  |
|  | Pre | 2.785 ± 0.204 | 2.739 ± 0.208 |  |  |  |  |  |
|  | Post | 2.738 ± 0.204 | 2.519 ± 0.208* |  | F-value: | 6,618 | 1,245 | 0,470 |
|  | 1h post | 2.211 ± 0.208 | 2.090 ± 0.208 |  | p-value: | **0,002** | 0,271 | 0,626 |
| PD1^+^ eT_regs_ [% CD4^+^] |  |  |  |  |  |  |  |  |
|  | Pre | 0.806 ± 0.057 | 0.782 ± 0.058 |  |  |  |  |  |
|  | Post | 0.611 ± 0.058 | 0.523 ± 0.058* |  | F-value: | 8.677 | 0.529 | 0.350 |
|  | 1h post | 0.749 ± 0.058 | 0.758 ± 0.058 |  | p-value: | **<0.001** | 0.471 | 0.705 |
| nT_regs_ [*10^6^/μl] |  |  |  |  |  |  |  |  |
|  | Pre | 0.993 ± 0.100 | 0.962 ± 0.105 |  |  |  |  |  |
|  | Post | 0.959 ± 0.102 | 0.723 ± 0.105* |  | F-value: | 20.660 | 2.077 | 1.254 |
|  | 1h post | 0.583 ± 0.105* | 0.496 ± 0.105* |  | p-value: | **<0.001** | 0.157 | 0.290 |
| nT_regs_ [% CD4^+^] |  |  |  |  |  |  |  |  |
|  | Pre | 0.387 ± 0.043 | 0.409 ± 0.038 |  |  |  |  |  |
|  | Post | 0.292 ± 0.039 | 0.214 ± 0.035*** |  | F-value: | 12.915 | 2.545 | 0.927 |
|  | 1h post | 0.315 ± 0.040 | 0.164 ± 0.032*** |  | p-value: | **<0.001** | 0.119 | 0.400 |
| IL-10 (pg/ml), *log_2_* |  |  |  |  |  |  |  |  |
|  | Pre | 4.454 ± 0.0763 | 4.454 ± 0.0763 |  |  |  |  |  |
|  | Post | 4.646 ± 0.0763 | 4.734 ± 0.0763* |  | F-value: | 16.399 | 1.965 | 1.414 |
|  | 1h post | 4.725 ± 0.0778* | 4.951 ± 0.0764*** |  | p-value: | **<0.001** | 0.168 | 0.248 |
| IL-6 (pg/ml), *log_2_* |  |  |  |  |  |  |  |  |
|  | Pre | 3.389 ± 0.1275 | 3.376 ± 0.1245 |  |  |  |  |  |
|  | Post | 3.859 ± 0.1304* | 3.813 ± 0.1273* |  | F-value: | 7.837 | 0.016 | 0.031 |
|  | 1h post | 3.709 ± 0.1337 | 3.722 ± 0.1273 |  | p-value: | **0.001** | 0.900 | 0.970 |
|  |  |  |  |  |  |  |  |  |

Data is presented as mean ± SEM. eT_regs_: effector T_regs_. HIIE: high-intensity interval exercise. MCE: moderate continuous exercise. MFI: median fluorescence intensity. nT_regs_: naive T_regs_. PD-1: programmed cell death protein 1. T_regs_: CD4^+^ regulatory T cells; ^*^different from baseline .005 < p ≤ .05. ^**^different from baseline .001 < p ≤ .005. ^***^different from baseline p ≤ .001

**Fig. S2** Connected individual data points of respective outcomes. The left part of **A**, **C**, and **D** represents the HIIE condition, whereas the right part represents the MCE condition. In **C**, pooled HIIE and MCE data are shown, since only main effects were detected.

**Fig. S3** Connected individual data points of respective outcomes. The left part of each figure represents the HIIE condition, whereas the right part represents the MCE condition.
